# Maternal and Adult Interleukin-17A Exposure and Autism Spectrum Disorder

**DOI:** 10.3389/fpsyt.2022.836181

**Published:** 2022-02-08

**Authors:** Masashi Fujitani, Hisao Miyajima, Yoshinori Otani, Xinlang Liu

**Affiliations:** Department of Anatomy and Neuroscience, Faculty of Medicine, Shimane University, Shimane, Japan

**Keywords:** maternal immune activation (MIA), IL-17A, autism spectrum disorder (ASD), Th17 cell, γδT cells, embryonic brain development, psoriasis

## Abstract

Epidemiological evidence in humans has suggested that maternal infections and maternal autoimmune diseases are involved in the pathogenesis of autism spectrum disorder. Animal studies supporting human results have shown that maternal immune activation causes brain and behavioral alterations in offspring. Several underlying mechanisms, including interleukin-17A imbalance, have been identified. Apart from the pro-inflammatory effects of interleukin-17A, there is also evidence to support the idea that it activates neuronal function and defines cognitive behavior. In this review, we examined the signaling pathways in both immunological and neurological contexts that may contribute to the improvement of autism spectrum disorder symptoms associated with maternal blocking of interleukin-17A and adult exposure to interleukin-17A. We first describe the epidemiology of maternal immune activation then focus on molecular signaling of the interleukin-17 family regarding its physiological and pathological roles in the embryonic and adult brain. In the future, it may be possible to use interleukin-17 antibodies to prevent autism spectrum disorder.

## Introduction

Prenatal exposure to maternal immune activation (MIA) has been implicated as an environmental risk factor for autism spectrum disorder (ASD). The relationship between MIA and the pathogenesis of neurodevelopmental disorders including ASD has been discussed at length (1–7).

In the first part of this review, we describe the epidemiology of MIA, including maternal infection and maternal autoimmune diseases, as risk factors for ASD. Subsequently, among immunological factors, we focus on molecular signaling of the interleukin (IL)-17 family regarding its physiological and pathological roles in the embryonic and adult brain, based essentially on animal experiments.

## Mia and Asd

Abnormalities in the immune system have been widely observed in the brain and periphery of patients with ASD. Studies have shown that ASD is associated with chronic neuroinflammation, with increased activation of microglia and astrocytes and the production of cytokines and chemokines in the brain (8, 9).

Infections during pregnancy can cause prematurity or stillbirth, and pathogens can be vertically transmitted to the fetus, causing congenital infections and severe diseases, known as TORCH syndrome (*Toxoplasma gondii*, other, rubella virus, cytomegalovirus, herpes simplex virus) (10, 11). In addition to the threat from these pathogens, other clinical evidence suggests that ASD is increased in the offspring of pregnancies during seasonal outbreaks and epidemics of influenza, measles, epidemic parotitis, and polio (7). Moreover, animal studies have shown that MIA, including viral infection and mimicry, results in neurodevelopmental abnormalities in rodents and non-human primates similar to human ASD phenotypes (3, 12, 13). However, this relationship has not been elucidated, because a meta-analysis showed that the odds ratio (OR) of offspring with ASD is only 1.13 (95% confidence interval [CI] 1.03–1.23) (14).

Furthermore, chronic inflammatory and allergic conditions in pregnancy, such as autoimmune diseases (15) (OR 1.34, 95% CI 1.23–1.46) or asthma (OR 1.43, 95% CI 1.38–1.49) (16), are prominent risk factors for ASD (6). The correlation between asthma and ASD has been well-demonstrated (17, 18). Among autoimmune diseases, maternal psoriasis is also a significant risk factor for ASD (OR 1.39, 95% CI 1.00–1.95) (18). Maternal psoriasis has recently received attention because IL-17A is one of the most important cytokines in the pathogenesis of psoriasis (19, 20).

## Il-17 Signaling

**Figure 1:**
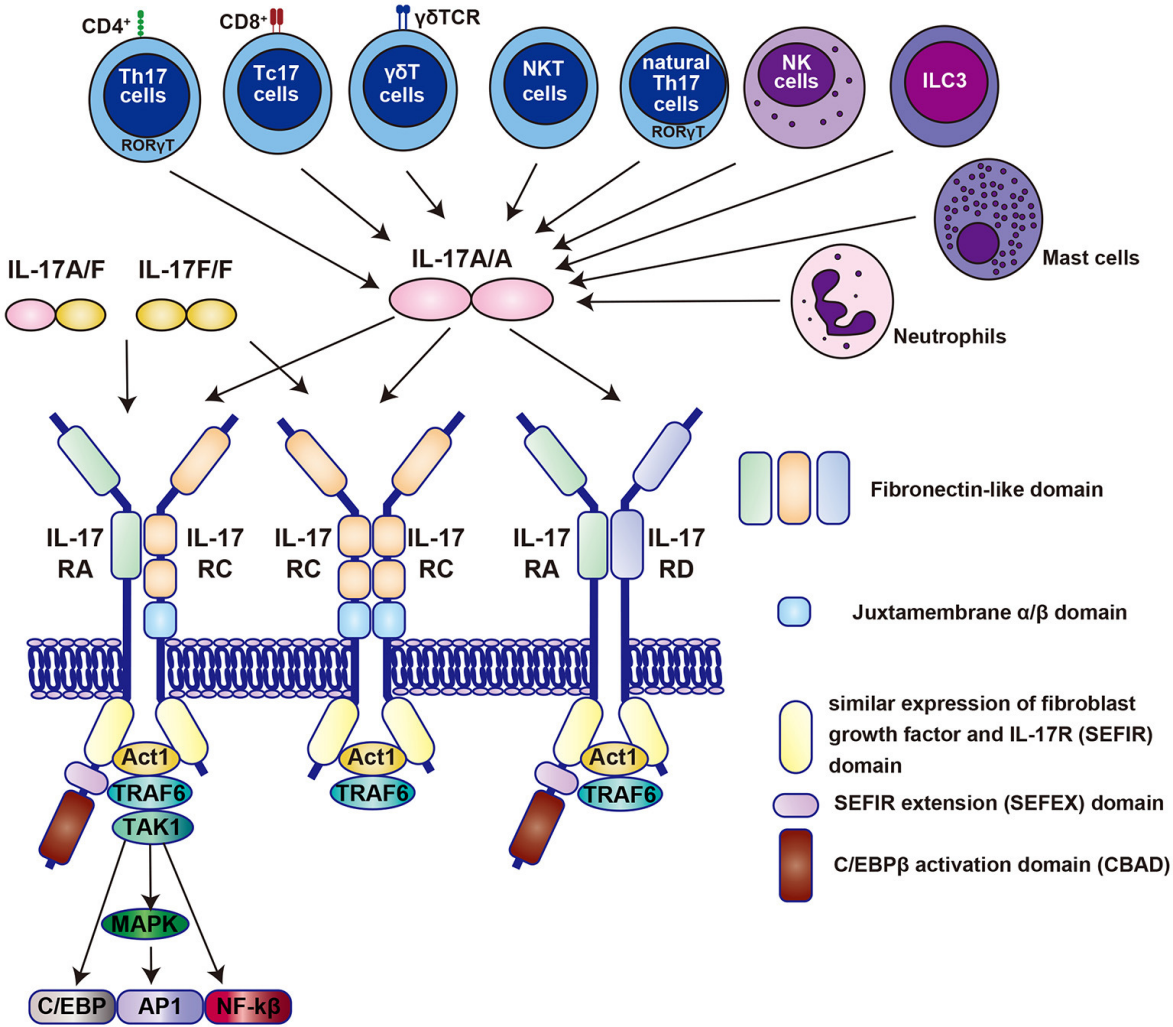
The molecular binding system of the IL-17 family centered on IL-17A and its receptors. All the family members of the IL-17 family, except IL-17D function as homodimer, whereas IL-17A and IL-17F form a heterodimer (denoted as IL-17A/F) (21). All receptors also function as homodimers or heterodimers. Homodimers of IL-17A (denoted as IL-17A/A) selectively bind to specific IL-17RA/RC, RC/RC, or RA/RD receptor complexes. Contrastingly, IL-17A/F and IL-17F/F bind only to IL-17RA/RC and RC/RC receptor complexes. Each IL-17 receptor has an extracellular fibronectin-like domain that binds the ligand and an intracellular SEFIR (similar expression of fibroblast growth factor and IL-17R) domain that recruits molecules such as Act1 and TRAF6 (21). The IL-17 receptor family has been shown by Goepfert et al., to be structurally bent between the first and second fibronectin domains (22). IL, interleukin; Th17, T helper 17; Tc17, IL-17-producing CD8^+^ T cells; NKT, natural killer T cells; NK; natural killer cells; ILC3, type 3 innate lymphoid cells; NF, nuclear factor; TRAF6, tumor necrosis factor receptor-associated factor 6; MAPK, mitogen-activated protein kinase; TAK1; transforming growth factor-β-activated kinase 1.

IL-17A (commonly known as IL-17) is a signature cytokine of a distinct CD4^+^ T helper 17 (Th17) cell that is characterized by the expression of retinoic acid receptor-related orphan receptor gamma t (RORγt) and is activated by IL-23. IL-17A is most strongly implicated in human disease among the six IL-17 family members (IL-17A, IL-17B, IL-17C, IL-17D, IL-25(also known as IL-17E), and IL-17F). As shown in Figure 1, all the family members except IL-17D basically function as homodimers; however, IL-17A and IL-17F form a heterodimer (21). The IL-23/IL-17A signaling axis has been found to play a critical role in autoimmune diseases (21, 23, 24).

It has been revealed that IL-17A is produced by other cell populations, such as IL-17-producing CD8^+^ T (Tc17) cells, γδT cells, natural killer T cells, natural Th17 cells, natural killer cells, group 3 innate lymphoid cells, neutrophils, and mast cells (21, 23, 24) (Figure 1).

The IL-17 receptor family is composed of five members (IL-17RA to IL-17RE), which are distinct subclasses of receptors characterized by an intracellular motif called SEFIR (SEF [similar expression to FGF receptor]/IL-17 receptor) (Figure 1) (21, 24). The initial event in IL-17R signaling is the recruitment of Act1, a multifunctional protein containing the SEFIR domain required for IL-17R-Act1 interaction. Act1 has E3 ubiquitin ligase activity and rapidly recruits and ubiquitinates tumor necrosis factor receptor-associated factor 6 (TRAF6), another E3 ubiquitin ligase (Figure 1). Like other receptors that recruit TRAF6, IL-17 triggers the activation of the canonical nuclear factor κB (NF-κB) cascade and pro-inflammatory and anti-microbial genes (21, 24). TRAF6 also promotes the activation of mitogen-activated protein kinase and activator protein 1 (AP1) pathways, and CCAAT/enhancer-binding protein (C/EBP) transcription factors (21, 24).

## Relationship Between Il-17 Exposure and Asd in the rodent Embryonic Brain

Little is known about the role of IL-17A in brain development under non-inflammatory conditions. Therefore, we obtained the RNA sequencing results from Mouse Genome Informatics and found that *Il-17a* and *Il-17f* were not or hardly expressed in the mouse embryonic brain (http://www.informatics.jax.org/). In contrast, IL-17 family receptors, *Il-17ra, rc, and rd*, are all expressed in the embryonic and adult mouse brain. These results were confirmed by Choi et al. in 2016, where they demonstrated that IL-17RA is expressed mainly in the cortical plate of the mouse embryonic brain (25).

**Figure 2:**
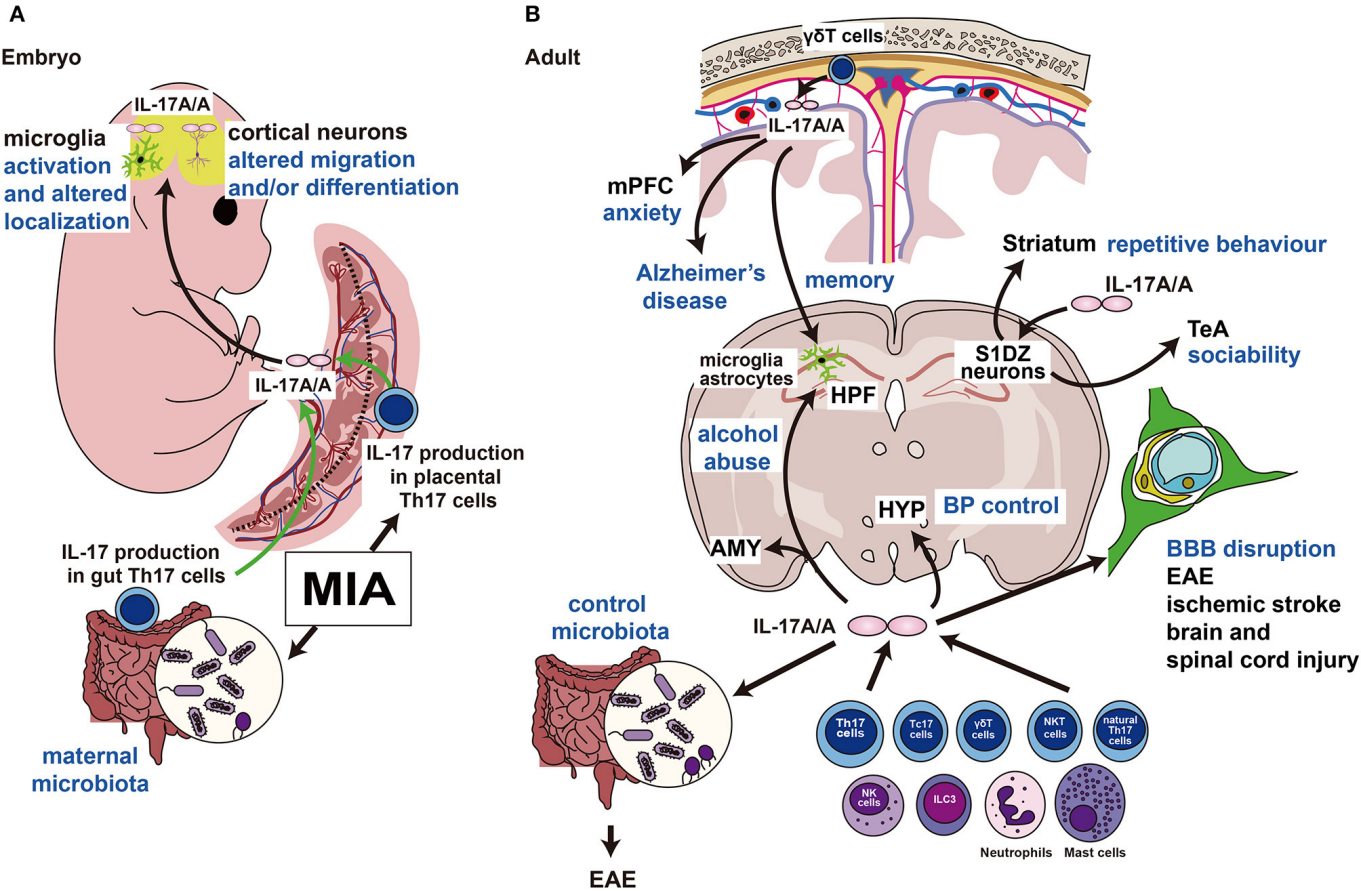
Functions of IL-17A in the embryonic and adult brains. **(A)** MIA induces the production of IL-17A (IL-17A/A indicates the homodimer) in Th17 cells of the maternal intestine and placenta. IL-17A crosses the blood-placental barrier and affects embryonic brain development. **(B)** IL-17A is produced by γδT cells in the cranial meninges and regulates the mPFC, which controls emotion, and HPF, which regulates memory formation. This pathway may be activated in the Alzheimer's disease model. Direct injection of IL-17A into the S1DZ restores the ASD phenotypes of repetitive behavior or sociability. IL-17A also controls the gut microbiota, and its disruption causes autoimmunity. IL17A is involved in the destruction of the BBB in several neurological disorders. Intravenous injection of IL-17A affects the hypothalamus and alters systemic blood pressure. IL, interleukin; MIA, maternal immune activation; Th17, T helper 17; mPFC; medial prefrontal cortex; HPF, hippocampal formation; S1DZ, primary somatosensory cortex dysgranular zone; ASD, autism spectrum disorder; BBB, blood-brain barrier; HYP, hypothalamus; BP, blood pressure; EAE, experimental allergic encephalomyelitis; Tc17, IL-17-producing CD8^+^ T cells; NKT, natural killer T cells; NK; natural killer cells; ILC3, type 3 innate lymphoid cells; AMY, amygdala; TeA, temporal association area.

Accumulating evidence supports a role for Th17 cells and their effector cytokine IL-17A in ASD (26) (Figure 2A). Polyinosinic:polycytidylic acid [poly (I:C)] is structurally similar to double-stranded RNA and is used to model the actions of extracellular double-stranded RNA, such as viral mimicry. Poly (I:C)-induced MIA in the gestating dam is relayed to the embryo via the placenta. Choi et al. (25) showed that increased Th17 cells in the placenta secrete IL-17A, which enters the fetal circulation. Since mouse blood brain barrier begins to form between E11 and E17 (27), circulated IL-17A can enter the brain and regulate development without local production of IL-17A (25). Another group also confirmed the upregulation of IL-17A in maternal blood and the postnatal offspring brain (28). In addition to poly (I:C), Lipopolysaccharide, major component of the outer membrane of Gram-negative bacteria, is also used to induce MIA as a bacterial septic shock model (29).

IL-17A acts directly on the mouse fetal brain on embryonic day (E) 14.5, resulting in an ASD-like phenotype, including abnormal behaviors in ultrasonic vocalization tests, social interaction tests, and marble burying tests (25). Direct injection of IL-17A into the fetal lateral ventricles on E14.5 resulted in phenocopied ASD-like behaviors and cortical disorganization in the offspring induced by poly (I:C)-evoked MIA (25). *Il-17ra* mRNA is detectable in the fetal brain on E14.5 and is upregulated by poly (I:C)-MIA in an IL-17A-dependent manner (25). Direct injection of IL-17A into the fetal brain on E14.5 resulted in thinning of the cortical plate on E18.5, which was not observed in MIA induction on E14.5 (25) (Figure 2A). Interestingly, Choi et al. found that poly (I:C)-induced MIA and IL-17A administration to the embryonic brain on E14.5 resulted in patch-like cortical dysplasia on E18.5 (25), which is similar to some human patients with ASD (30). Their group reproduced the results by another study (31); however, another group mentioned that they could not find any patches after MIA; therefore, the occurrence of cortical patches remains controversial (32).

Kim et al. showed that maternal microbiota, including segmented filamentous bacteria (SFB), promote IL-17A production in maternal gut Th17 cells (33). They treated MIA-evoked dams with vancomycin to kill SFB, and this treatment inhibited the ASD-phenotype in offspring, such as abnormal ultrasonic vocalization, repetitive behavior, or sociability, with decreased IL-17A production (Figure 2A). More recently, another group showed that the administration of IL-17A during the entire maternal period causes early and persistent cortical abnormalities and ASD-like phenotypes in male offspring (34). The offspring showed abnormal expression of synaptic and cell cycle genes, disrupted adult glia, inhibitory synapses, and abnormal behaviors (34). Moreover, IL-17A injection into the fetal brain on E14.5 resulted in microglial activation and altered localization (35) (Figure 2A). In addition, maternal overexpression of IL-17A induced abnormal behavior in offspring, and in parallel, elevated kynurenine levels in maternal serum and fetal plasma were observed. Moreover, maternal kynurenine-injected mice exhibited behavioral abnormalities similar to those observed in the offspring of *Il-17a*-overexpressed dams (36) (Figure 2A).

## Il-17A Exposure in the Adult Brain of Rodents

Contrary to the analysis in the embryonic brain, the expression of IL-17A and its receptors in the adult central nervous system (CNS) has been intensively studied. Das Sarma et al. showed that IL-17RA is expressed in some cultured astrocytes (16.8%) and slightly in microglia (0.80%) (37), and Liu et al. (2014) showed that in the adult dentate gyrus, astrocytes mainly express *Il17a* under physiological conditions (38). Their study revealed that IL-17A is a negative regulator of neurogenesis in the adult hippocampus, and *Il17a* knockout enhances synaptic function (38).

In addition to these published results, we obtained the RNA sequencing results from the Human Brain Atlas (https://www.proteinatlas.org/) and Brain RNA-Seq (https://www.brainrnaseq.org/), based on published papers (39, 40). According to these databases, *Il-17a* and *Il-17f* mRNA are rarely expressed in any cell type in the mouse brain; *Il-17ra* mRNA is mainly expressed in macrophages/microglia in small amounts in oligodendrocytes, neurons, and oligodendrocyte precursor cells and is almost absent in astrocytes and endothelial cells. In terms of tissue distribution, a small amount of *Il-17ra* mRNA was observed in the cerebral cortex. Since *Il-17rc* and *rd* mRNA are much more abundant in the pituitary gland, it is necessary to analyze the expression of each isoform of the IL-17 receptor.

Chen et al. used forward genetic methods to show that the *Caenorhabditis elegans* homolog of *Il-17a* functions as a neuromodulator in somatosensory neurons (41). Subsequently, Ribeiro et al. showed that IL-17A controls synaptic plasticity and short-term memory (42) (Figure 2B). Intriguingly, IL-17A is secreted by fetal-derived meningeal resident γδT cells and plays an important role in memory formation via glial cell production of brain-derived neurotrophic factor under physiological conditions (42). Furthermore, even under physiological conditions, IL-17A secreted from γδT cells and IL-17RA signaling in neurons of the medial prefrontal cortex controls anxiety-like behaviors, not sociability or memory (43). Alves De Lima et al. also found that the number of meningeal γδT cells increases after birth; therefore, depletion of IL-17A or γδT cells in the postnatal period may affect behavior (43) (Figure 2B).

Reed et al. showed the beneficial effects of IL-17A on social behavior disorders (44) (Figure 2B). They first detected abnormalities in the neural circuits responsible for repetitive behavior and sociability examined using the marble burying test and social interaction test, respectively (31). The main focus of abnormal circuits in MIA offspring is the primary somatosensory cortex dysgranular zone (S1DZ). Interestingly, using optogenetics, it was reported that S1DZ neurons projecting to the temporal association cortex control sociability, and S1DZ neurons projecting to the striatum regulate repetitive behavior in MIA offspring (31). On the other hand, lipopolysaccharide administration can restore social behavioral deficits in MIA-exposed offspring. More interestingly, direct IL-17A delivery into the S1DZ can also restore disturbed social behavior even in monogenic ASD mouse models such as *Cntnap2* or *Fmr1* mutant mice (44). The authors concluded that the production of IL-17A during inflammation can ameliorate the expression of social behavior deficits by directly affecting neural activity in the brain (44).

In addition to ASD, IL-17A signaling has received strong attention for its pathophysiological functions in various neurological disorders (45–48) (Figure 2B). In particular, the importance of IL-17A has been strongly demonstrated in experimental allergic encephalomyelitis (EAE), a model of multiple sclerosis (45, 47, 48). In a recent study, *Il-17a/f-*deficient mice lost sensitivity to EAE, which correlates with changes in the gut microbiota (49). Another important aspect of IL-17A is the regulation of blood-brain barrier functions (50–52) (Figure 2B). Furthermore, the involvement of IL-17A signaling has been revealed in various experimental models of ischemic brain injury (53), traumatic brain injury (54), and spinal cord injury (55, 56) (Figure 2B).

In addition to the immunological disorders described above, emerging evidence suggests that IL-17A secreted by meningeal γδT cells regulates the pathogenesis of Alzheimer's disease (57, 58), IL-17A secreted by Th17 cells is involved in alcohol abuse (59), and IL-17A regulates blood pressure via the activation of paraventricular nucleus neurons (60) (Figure 2B).

## Relationship Between Il-17A Exposure in the Human Brain and Asd

It has been reported that neurons, glia, and endothelial cells in the human cortex express receptors for IL-17 (61). However, no information on the specific expression of the IL-17 receptor isoform in the human brain has been reported. Therefore, we obtained the RNA sequencing results from the Human Protein Atlas (https://www.proteinatlas.org/) and Brain RNA-Seq (https://www.brainrnaseq.org/), based on a published paper (62). We found that *IL17A* is rarely expressed in the human brain as revealed by both databases. On the other hand, receptors for the IL-17 family, *IL-17RA* and *RC*, are both expressed in embryonic and adult brains as examined by both databases.

In some patients with ASD, IL-17A has been found at high levels in the blood and correlates with the severity of behavioral symptoms (63, 64). A genome-wide association study showed that copy number variation of the *IL17A* gene is a risk factor for ASD (65). However, the evidence indicated an indirect correlation. Therefore, to show a direct causal relationship between maternal IL-17A exposure and ASD, we propose the following clinical investigation.

**Figure 3:**
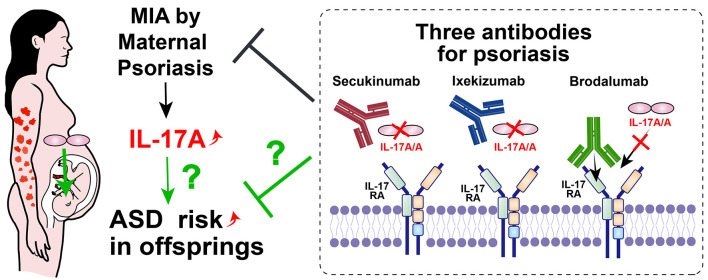
Possible ASD prevention method by modulating MIA with IL-17 related antibodies. Maternal psoriasis is one of the risk factors for offspring ASD. Three available antibodies against IL-17A or IL-17RA for psoriasis, psoriatic arthritis and ankylosing spondylitis could potentially be used for maternal patients. If the risk of ASD was reduced by clinical studies with these antibodies, the direct relationship between MIA and ASD through IL-17 signaling will clearly be revealed.

First, psoriasis has received much attention in recent years, since maternal psoriasis is also a significant risk factor for ASD (18), and IL-17A is one of the most important cytokines in the pathogenesis of psoriasis (19, 20). Therefore, maternal psoriasis is a candidate disease to be investigated (Figure 3).

To modulate IL-17A signaling, three commercially available antibodies are currently available to treat humans: secukinumab (human monoclonal antibody to IL-17A, immunoglobulin [Ig]G1), ixekizumab (humanized monoclonal antibody to IL-17A, IgG4), and brodalumab (human monoclonal antibody to the IL-17 receptor, IgG2). Both secukinumab and ixekizumab are approved for psoriasis, psoriatic arthritis, and ankylosing spondylitis; brodalumab is only approved for the treatment of psoriasis (66). All subclasses of IgG (IgG1–IgG4) cross the human placenta (67), therefore, all candidate antibodies can block the abnormal upregulation of IL-17 signaling in the fetus. Since the human blood brain barrier also begins to form during pregnancy as well as in mice (68), candidate antibodies may enter the fetal brain after angiogenesis. Clinically, no complication with prenatal usage of secukinumab was reported (69).

If the incidence of ASD is reduced in the offspring of pregnant patients with psoriasis treated with IL-17-related antibodies, this would indicate a direct causal relationship between MIA and ASD via IL-17 signaling (Figure 3).

Lastly, considering the current pandemic situation, I can't avoid mentioning the topic of COVID-19. To the best our knowledge, no evidence that COVID-19 in pregnant mother could be the risk of ASD in offspring has been reported. However, some reports showed that IL-17A is involved in pathophysiology of COVID-19 infection (70), therefore long term observation will elucidate whether maternal COVID-19 infection may impact fetal brain development (71).

## Discussion

The pathophysiological mechanism of ASD or brain development caused by MIA or IL-17A exposure remains to be addressed. As mentioned in section Relationship Between IL-17A Exposure in the Human Brain and ASD, in humans, there is a lack of direct causal relationship between IL-17A and ASD.

Even in experimental animals, the following questions remain elusive. First, MIA has been shown to cause abnormalities in fetal brain development, including unexplained cortical dysgenesis. Wong et al. found that adult offspring exposed to MIA on E14.5 had significantly reduced numbers of either TBR1^+^ or SATB2^+^ cells in the cortex with cortical patches (26) [see also Shin Yim et al. (31)]. During cortical development, exposure to MIA and IL-17A transiently delays the production of SATB2^+^ cells on E14.5 and alters cortical neurogenesis or radial migration only at the medial area with cortical patch formation without changing cortical thickness. Furthermore, IL-17RA is only expressed in cortical plates, and the cell type is unknown (26). These phenotypes cannot be explained by abnormalities in neurogenesis or radial migration of the entire radial glia. Another study suggested that microglia may alter the neurogenesis of radial glia or neural migration, as IL-17A injection induces microglia to migrate closer to the lateral ventricles (35). In support of this idea, microglia, but not neurons or other glial types, express the highest amount of *Il-17ra* in the adult stages as examined by databases. Second, it has been suggested that MIA can affect brain development into adulthood with altered systemic immunological responses (30, 72). It has long been unclear why these effects persist, but recent evidence might answer this question. Lim et al. infected pregnant mice with *Yersinia pseudotuberculosis*. Although the infection was restricted to the dam, the offspring surprisingly harbored more intestinal Th17 cells into adulthood via IL-6 signaling (73). Maternal IL-6 induced immediate and long-term effects based on changes in the epigenetic memory of fetal intestinal epithelial stem cells. Therefore, an enhanced response to the microbiota is trained during pregnancy, and the immune response system is already altered at birth (73).

In this review, we summarize how IL-17A affects brain development and adult brain function mostly based on the animal experiments. In the near future, it may be possible to use IL-17A related antibodies to prevent ASD. However, the involvement of IL-17A signaling has not been elucidated yet. Future clinical studies will help to answer this question.

## Author Contributions

MF: conceptualization and writing—original draft preparation. HM, YO, and XL: writing—review, editing and visualization. All authors contributed to the article and approved the submitted version.

## Funding

This research was funded by the Japan Society for the Promotion of Science, JSPS, through 21K16589 and 20K22952 for HM. This research was funded by the Osaka Medical Research Foundation for Intractable Diseases and the Ichiro Kanehara Foundation for the Promotion of Medical Sciences and Medical Care for YO. This research was funded by Shimane Prefecture Technology seeds supporting project: 2021 for MF.

## Conflict of Interest

The authors declare that the research was conducted in the absence of any commercial or financial relationships that could be construed as a potential conflict of interest.

## Publisher's Note

All claims expressed in this article are solely those of the authors and do not necessarily represent those of their affiliated organizations, or those of the publisher, the editors and the reviewers. Any product that may be evaluated in this article, or claim that may be made by its manufacturer, is not guaranteed or endorsed by the publisher.

## Abbreviations

ASD: autism spectrum disorder
CI: confidence interval
CNS: central nervous system
E: embryonic day
EAE: experimental allergic encephalomyelitis
Ig: immunoglobulin
IL: interleukin
MIA: maternal immune activation
OR: odds ratio
S1DZ: primary somatosensory cortex dysgranular zone
SEFIR: similar expression to FGF receptor /IL-17 receptor
SFB: segmented filamentous bacteria
Th17: T helper 17
TRAF6: tumor necrosis factor receptor-associated factor 6.
